# Evaluation of bovine (*Bos indicus*) ovarian potential for *in vitro* embryo production in the Adamawa plateau (Cameroon)

**Published:** 2014-12-28

**Authors:** J. Kouamo, S.M. Dawaye, A.P. Zoli, G.S. Bah

**Affiliations:** 1 *School of Veterinary Medicine and Sciences, University of Ngaoundere, P.O. BOX: 454, Ngaoundere, Cameroon*; 2 *Regional Center of the Institute of Agricultural Research for Development (IRAD) Wakwa. P.O. BOX 65, Ngaoundere, Cameroon*

**Keywords:** Adamawa breed, Embryo, Follicular population, Oocyte quality, Ovary

## Abstract

An abattoir study was conducted to evaluate the ovarian potential of 201 local zebu cattle from Ngaoundere, Adamawa region (Cameroon) for *in vitro* embryo production (IVEP). The ovaries were excised, submerged in normal saline solution (0.9%) and transported to the laboratory for a detailed evaluation. Follicles on each ovary were counted, their diameters (Φ) measured and were grouped into 3 categories: small (Φ < 3 mm), medium (3 ≥ Φ ≤ 8 mm) and large (Φ > 8 mm). Each ovary was then sliced into a petri dish; the oocytes were recovered in Dulbecco’s phosphate buffered saline, examined under a stereoscope (x10) and graded into four groups based on the morphology of cumulus oophorus cells and cytoplasmic changes of the oocytes. Grade I (GI): oocytes with more than 4 layers of bunch of compact cumulus cells mass with evenly granulated cytoplasm; grade II (GII): oocyte with at least 2-4 layers of compact cumulus cell mass with evenly granulated cytoplasm; grade III (GIII): oocyte with at least one layer of compact cumulus cell mass with evenly granulated cytoplasm; grade IV (GIV): denuded oocyte with no cumulus cells or incomplete layer of cumulus cell or expanded cells and having dark or unevenly granulated cytoplasm. The effects of both ovarian (ovarian localization, corpus luteum, size and weight of ovary) and non-ovarian factors (breed, age, body condition score (BCS) and pregnancy status of cow) on the follicular population and oocyte recovery rate were determined. There were an average of 16.75±0.83 follicles per ovary. The small, medium and large follicles were 8.39±0.60, 8.14±0.43 and 0.21±0.02 respectively. Oocyte recovery was 10.97±0.43 per ovary (65%). Oocytes graded I, II, III and IV were 3.53±0.19 (32.21%), 2.72±0.15 (24.82%), 2.24±0.15 (20.43%) and 2.47±0.20 (22.54%) respectively. The oocyte quality index was 2.26. Younger non pregnant cows having BCS of 3 and large ovaries presented higher number of follicles and oocyte quality (P < 0.05) compared with other animals. Oocytes with quality (grade I and II) acceptable for IVEP constituted 57.15% of the harvest. This study indicated that factors such as age, pregnancy status, BCS and ovarian size must be taken into account to increase the potential of the ovary for IVEP.

## Introduction

Cattle production occupies an important place in Cameroon’s national animal production systems. With 7 million cattle, they represent 10% of the total livestock (MINEPIA, 2009). The cattle raised in Cameroon are mostly zebus (*Bos indicus*) (Sartori and Barros, 2011) which are trypano-susceptible. However, the *Bos Taurus* cattle (Namchi, Kapsiki, Kuri or Bakosi) are very robust and trypano-tolerant (Donelson, 2003) and constitutes a relatively small (2%) proportion of the total cattle population and are considered to be highly endangered breeds (Lhoste, 1991).

The main physiological differences between *Bos indicus* cattle and *Bos taurus* cattle include: delayed age at puberty (Rodrigues *et al.*, 2002); higher circulating concentrations of hormones such as estradiol, progesterone, insulin and IGF-I (Bastos *et al.*, 2010), despite having a smaller ovulatory follicle size and corpora lutea (Bastos *et al.*, 2010); greater population of small follicles (Gimenes *et al.*, 2009) and smaller size of the dominant follicle at deviation (Gimenes *et al.*, 2008); and greater sensitivity of follicles to gonadotropins (Randel, 1984).

Despite the rich breed diversity of local cattle, their productivity still remains low. Genetic, husbandry, health and reproductive problems have previously been identified as factors responsible for the low cattle productivity (Ebangi *et al.*, 2011).

The local cattle are bred naturally under traditional management systems with very little or no breeding programs. Artificial insemination (AI) was introduced for the first time in Africa in 1935 in Kenya and was widespread throughout sub-Saharan Africa in support of various improvement projects (Kouamo *et al.*, 2009).

In 1934, the first cattle improvement project in Cameroon was initiated at Wakwa, the Adamawa region by Pierre Blanc, a French veterinarian who was the first to train Cameroonian inseminators in April 1944. The calving rate after the first AI trial was 30.16% (Mandon, 1948). Currently, AI is rarely used. Certain cattle breeders in the Adamawa region artificially inseminate their local zebu cattle with imported exotic dairy semen in an unregulated manner thereby causing dispersion and dilution of protected local genotype (Bah *et al.*, 2010).

*In vitro* Embryo Production (IVEP) and Embryo Transfer (ET) are reproductive techniques that supplement AI in the genetic improvement of local cattle breeds (Hernandez-Fonseca *et al.*, 2002). IVEP permits the preservation of genetic potential of sub-fertile or dead animals (Deuleuze *et al.*, 2009) by the creation of a gene bank with oocytes recovered from slaughterhouses (Seidel and Seidel, 1989) for the improvement of livestock productivity (Huang and Rosenwarks, 2012).

Samples collected from slaughterhouse are the cheapest and the most abundant source of primary oocytes for large scale production of embryos through *in vitro* maturation (IVM) and *in vitro* fertilization (IVF) (Nandi *et al.*, 2006). The initial and the most important step in IVF is the selection of viable oocytes for IVM (Kouamo and Kharche, 2014). In Sub-Sahelian Africa, oocyte recovery rate is poor and the cost of IVEP high (Kouamo *et al.*, 2009).

To our knowledge, such study has never been conducted in Cameroon. Therefore, the current study was carried out to evaluate the ovarian potential of local zebu cattle for IVEP in Cameroon. Specific objectives were to characterize the slaughtered cows and their ovaries, to determine the follicular population and oocyte recovery rate and evaluate the effects of ovarian and non-ovarian factors on follicular population and oocytes recovery.

## Materials and Methods

### Study Area

The study was conducted using samples collected at the Ngaoundere Municipal Slaughterhouse (NMSH) and analyzed at the Veterinary laboratory of IRAD-Wakwa Regional Center (Physiology and reproduction biotechnology department) in Adamawa region of Cameroon.

The cattle slaughtered at the NMSH were from the Vina Division (61%) and Mayo Rey Division (39%). Ngaoundere is situated between Latitude 7°19’39N and Longitude 13°35’4E and have an average annual rainfall of 1496.7 mm. The temperatures varied from 15.2°C to 29°C with an average humidity of 58.2%. The study was conducted from November, 2013 to March, 2014.

### Characteristics of animals

A total of 201 local cows of different breeds [Gudali (92), White Fulani (58), Red Fulani (31) and Bokolo (20)] were randomly selected for this study. The mean live weight [estimated from thoracic circumference (THC) as follows (124.69 - 3.171 x + 0.0276 x THC x THC²) (Njoya *et al.*, 1997)], body condition score (BCS) as described by Natumanya *et al*. (2008) and age as described by Lucyna and Zdzisław (1984) have been determined. Fetal age was determined by the formula Y = X (X + 2), X represented the number of months of pregnancy and Y the crown-rump length in centimeters (Santos *et al.*, 2013) and the stage of pregnancy was classified as first (≤90 days), second (91-180 days) and third trimester (>180 days).

### Ovary collection and handling

After slaughter, the left and right ovaries were excised and placed in separate conical tubes containing Washed Medium (WM) and transported to the laboratory at 35-37°C within next 2 hours after slaughter. All cystic ovaries were excluded from studies (Wang *et al.*, 2007).

### Determination of the weight and the size of the ovary

In the laboratory, excessive tissues attached to the ovaries were carefully trimmed off and ovaries were weighed using an electronic scale, Mettler PC 2000. The length, width and thickness of the ovaries were measured using electronic Vernier calipers and the ovaries were thereafter allocated into two size groups (<2.25×1.75×1.25 and >2.25×1.75×1.25) as described by Samad and Raza (1999).

### Determination of follicular population

The ovaries were washed with WM. For each ovary, visible follicles were counted and follicular Φ was measured with electronic Vernier calipers. Follicular Φs were classified into 3 categories: small (<3 mm), medium (3 to 8 mm) and large (> 8 mm) as described by Baki Acar *et al*. (2013).

### Recovery and grading of oocytes

The ovaries were placed in separate plastic Petri dishes containing Dulbecco’s Phosphate Buffered Saline (DPBS) and chopped into small pieces with a scalpel blade (slicing) to release the oocytes (Wang *et al.*, 2007).

Oocyte quality was evaluated under a stereoscope (x10) and scored into four grades (G) according to the homogeneity of the cytoplasm and layers of cumulus cells as described by Alves *et al*. (2014).

Grade I (GI): Oocytes with more than 4 layers of bunch of compact cumulus cells mass with evenly granulated cytoplasm; grade II (GII): oocyte with at least 2–4 layers of compact cumulus cell mass with evenly granulated cytoplasm; grade III (GIII): Oocyte with at least one layer of compact cumulus cell mass with evenly granulated cytoplasm; grade IV (GIV): Denuded oocyte with no cumulus cells or incomplete layer of cumulus cell or expanded cells and having dark or unevenly granulated cytoplasm. Their overall quality was calculated as an index using the formula [(G I × 1 + G II × 2 + G III x 3 + G IV × 4) / Total number of oocytes recovered] as described by Baki Acar *et al*. (2013). Index values that approache one reflected good quality oocytes.

### Statistical analysis

Data were analyzed using SPSS (Statistical Package for Social Sciences) Version 20. The analysis of variance and Duncan’s test statistics were used to analyze appropriate data sets. Differences were significant at P < 0.05.

## Results

### Characterization of cows and ovaries

The mean live weight (Kg), BCS and age (years) of the cows were 382.08±70.73; 2.67±0.06 and 6.80±0.15, respectively. The mean weight (g) of the ovaries was 4.60±1.82. The right ovaries (4.99±2.48 g) were heavier than the left (4.22±2.15 g).

The length, wide and thickness (cm) of the ovaries were 2.78±0.55, 1.90±0.42 and 1.27±0.33, respectively.

The ovaries of pregnant cows were larger and heavier than those of non-pregnant ones (P<0.05). Also cows with BCS of 3 had heavier ovaries than others. The length of the ovaries increased significantly with age (Table 1).

**Table 1:** Means (±SE) values of the weight and size of the ovaries, breed, BCS, age, pregnancy status and corpus luteum.

Factors		N	Right ovary weight (g)	Left ovary weight (g)	Ovary weight (g) per animal	Ovary length (cm)	Ovary width (cm)	Ovary thickness (cm)
Breed	White Fulani	58	5.07±0.32^a^	4.20±0.26^a^	4.63±0.23^a^	2.79±0.06^a^	1.90±0.04^a^	1.27±0.03^a^
	Bokolo	20	4.99±0.87^a^	3.41±0.36^a^	4.20±0.27^a^	2.68±0.08^a^	1.91±0.05^a^	1.21±0.55^a^
	Red Fulani	31	4.62±0.58^a^	4.56±0.42^a^	4.59±0.42^a^	2.82±0.09^a^	1.89±0.06^a^	1.29±0.05^a^
	Gudali	92	5.05±0.24^a^	4.28±0.23^a^	4.67±0.18^a^	2.77±0.05^a^	1.90±0.03^a^	1.30±0.03^a^
	P-value		0.546	0.675	0.776	0.767	0.998	0.565
BCS	Thin (1-2)	81	4.35±0.24^a^	3.78±0.22^a^	4.06±0.18^a^	2.68±0.05^a^	1.81±0.03^a^	1.18±0.02^a^
	Good (3)	93	5.30±0.26^b^	4.45±0.24^b^	4.87±0.26^b^	2.85±0.06^a^	1.96±0.03^b^	1.34±0.03^b^
	Fat (4-5)	27	5.82±0.55^b^	4.70±0.37^b^	5.26±0.31^b^	2.82±0.06^a^	1.97±0.04^b^	1.36±0.05^b^
	P-value		0.007	0.027	0.002	0.066	0.002	0.002
Age (Years)	3-5	55	4.79±0.28^a^	3.87±0.22^a^	4.33±0.19^a^	2.58±0.05^a^	1.85±0.03^a^	1.30±0.03^a^
	6-9	108	5.12±0.25^a^	4.28±0.21^a^	4.70±0.18^a^	2.84±0.05^b^	1.92±0.03^a^	1.27±0.02^a^
	10-15	38	4.87±0.45^a^	4.52±0.42^a^	4.69±0.33^a^	2.86±0.08^b^	1.89±0.06^a^	1.29±0.07^a^
	P-value		0.923	0.063	0.441	0.003	0.302	0.763
Pregnancy status	Non pregnant	96	4.26±0.20^a^	3.54±0.18^a^	3.90±0.16^a^	2.62±0.04^a^	1.79±0.03	1.18±0.02
	Pregnant	105	5.65±0.26^b^	4.84±0.22^b^	5.24±0.17^b^	2.92±0.05^b^	2.00±0.03^b^	1.37±0.02^b^
	P-value		0.000	0.000	0.000	0.000	0.000	0.000
CL	Absent	61	3.57±0.18^a^	3.22±0.16^a^	3.39±0.16^a^	2.60±0.05^a^	1.69±0.03^a^	1.09±0.02^a^
	Present	140	5.60±0.22^b^	4.22±0.15^b^	5.12±0.15^b^	2.85±0.04^b^	1.99±0.02^b^	1.36±0.02^b^
	P-value		0.000	0.009	0.000	0.001	0.000	0.000

^a,b,c^ In each column different letters (a, b) indicated significant difference between group (p<0.05). N=number of cows. SE=standard error

### Follicular population

From 402 ovaries, 6,747 follicles were counted. The mean number of follicles per ovary recorded was 16.75±0.83. Small (Φ < 3mm), medium (3 ≥ Φ ≤ 8 mm) and large (Φ > 8 mm) follicles represented 50.12%, 48.63% and 1.25% of the follicular population, respectively.

### Oocyte recovery rate

**Fig. 1:**
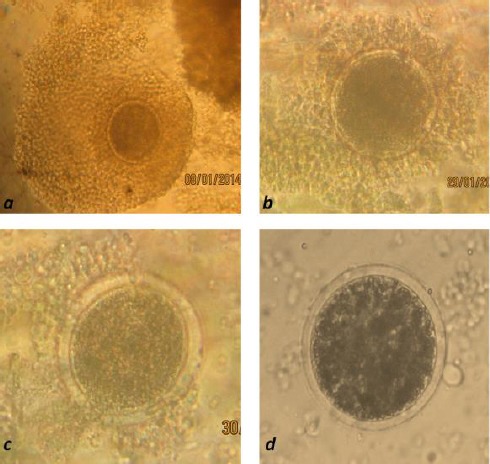
a= Oocyte grade I; b= Oocyte grade II; c= Oocyte grade III; d= Oocyte grade IV.

The overall average oocytes recovered per ovary was 10.97±0.43 (n = 4,411) with a recovery rate of 65%. The quality of the oocytes graded I, II, III and IV (Figure 1) were 3.53±0.19 (32.21%), 2.72±0.15 (24.82%) 2.24±0.15 (20.43%) and 2.47±0.20 (22.54%) respectively. Selected oocytes for IVEP (G I and II) represented 6.27±0.32 (57.15%) per ovary. The oocyte index was 2.26.

### Effect of ovarian factors (ovarian localization, corpus luteum, size and weight of ovary) on the follicular population, oocyte number and grade

The right ovary tended to have more follicles and oocytes than the left (P>0.05). The yield and quality of oocytes from follicles increased with the weight (g) and size (cm) of the ovaries. The ovaries with CL had more medium follicles (Tables 2a and 2b).

**Table 2a:** Effects of ovarian factors on follicular population.

Factors		N	Number of follicles			Average number of follicles /ovary
			Small (< 3 mm)	Medium (3-8 mm)	Large (>8 mm)
Ovary localization	Right	201	8.24±0.62^a^	8.45±0.47^a^	0.25±0.04^a^	16.95±0.86^a^
	Left	201	8.55±0.64^a^	7.83±0.48^a^	0.18±0.03^a^	16.57±0.88^a^
	P-value					0.756
CL	Absent	260	8.99±0.57^a^	7.31±0.38^a^	0.23±0.03^a^	16.53±0.73^a^
	Present	142	7.31±0.70^a^	9.68±0.64^b^	0.18±0.03^a^	17.17±1.12^a^
	P-value					0.623
Ovary weight (g)	<3	134	5.33±0.52^a^	5.74±0.49^a^	0.25±0.04^a^	11.32±0.79^a^
	3-5	134	7.55±0.65^b^	7.94±0.53^b^	0.24±0.04^a^	16.47±0.88^b^
	>5	134	11.41±0.99^c^	10.75±0.63^c^	0.16±0.03^a^	22.49±1.26^c^
	P-value					0.000
vary size (cm)	<2,25×1,75×1,25	142	5.07±0.40^a^	6.21±0.40^a^	0.29±0.05^a^	11.57±0.60^a^
	>2,25×1,75×1,25	174	10.81±0.79^b^	10.72±0.60^b^	0.18±0.03^a^	21.71±1.10^b^
	P-value					0.000

^a,b,c^ In each column different letters (a, b) indicated significant difference between group (p<0.05). N=number.

**Table 2b:** Effects of ovarian factors on oocyte number and grade.

Factors		N	Average number of oocytes /ovary	Oocyte grades				Selected oocytes for IVEP. I and II (%)
				I	II	III	IV	
Ovary localization	Right	201	11.28±0.62^a^	3.55±0.22^a^	2.83±0.18^a^	2.30±0,17^a^	2.60±0.26^a^	6.38±0.36^a^(56.56)
	Left	201	10.67±0.60^a^	3.52±0.21^a^	2.62±0.18^a^	2.17±0,17^a^	2.36±0.19^a^	6.13±0.35^a^(57.45)
	P-value		0.756					0.631
CL	Absent	260	11.51±0.53^a^	3.58±0.19^a^	2.86±0.16^a^	2.37±0,15^a^	2.70±0.21^a^	6.44±0.31^a^(55.95)
	Present	142	9.99±0.75^a^	3.45±0.28^a^	2.47±0.19^a^	2.08±0,19^a^	2.07±0.24^a^	5.92±0.43^a^(59.25)
	P-value		0.095					0.330
Ovary weight (g)	<3	134	8.44±0.59^a^	2.66±0.22^a^	2.09±0.22^a^	1.78±0,18	1.90±0.21	4.75±0.35^a^(56.27)
	3-5	134	11.39±0.74^b^	3.51±0.24^b^	3.51±0.24^b^	2.29±0,20	2.59±0.33	6.50±0.44^b^(57.07)
	>5	134	13.09±0.84^b^	4.43±0.32^b^	2.64±0,23	2.64±0,23	2.93±0.28	7.51±0.48^b^(57.37)
	P-value		0.000					0.000
Ovary size (cm)	<2,25×1,75×1,25	142	8.57±0.57^a^	2.65±1.93^a^	2.15±0.17^a^	1.68±0,17^a^	2.08±0.22^a^	4.80±0.33^a^(56.00)
	>2,25×1,75×1,25	174	12.57±0.73^b^	4.14±0.26^b^	3.16±0.21^b^	2.58±0.19^b^	2.69±0.23^a^	7.30±0.42^b^(58.07)
	P-value		0.000					0.000

^a,b,c^ In each column different letters (a, b) indicated significant difference between group (p<0.05). N=number.

### Effect of non-ovarian factors (breed, age, BCS, state and pregnancy length) on the follicular population, oocyte number and grade

Follicular population and oocyte recovery rate were higher in cows aged less than 10 years with a BCS of 3, not pregnant or carrying a fetus in the first trimester of pregnancy (P<0.05; Tables 3a and 3b).

**Table 3a:** Effects of non-ovarian factors on follicular population.

Factors		N	Number of follicles			Average number of follicles /ovary
			Small (< 3 mm)	Medium (3-8 mm)	Large (>8 mm)	
Breed	White Fulani	58	9.60±1.20^a^	7.63±0.91^a^	0.19±0.03^a^	17.39±1.74^a^
	Bokolo	20	7.80±2.04^a^	8.00±1.26^a^	0.25±0.07^a^	16.02±2.45^a^
	Red Fulani	31	9.10±1.83^a^	8.22±1.03^a^	0.22±0.06^a^	17.47±2.42^a^
	Gudali	92	7.53±0.76^a^	8.48±0.62^a^	0.20±0.04^a^	16.27±1.10^a^
	P-value					0.170
Age (years)	3-5	55	8.52±1.08^a^	8.55±0.67^a^	0.34±0.05^a^	17.42±1.38^a^
	6-9	108	8.61±0.86^a^	9.14±0.59^a^	0.17±0.02^b^	17.76±1.13^a^
	10-15	38	7.59±1.34^a^	4.74±1.06^b^	0.13±0.04^b^	12.46±2.16^b^
	P-value					0.043
BCS	Thin (1-2)	81	7.61±0.79^a^	6.68±0.63^a^	0.20±0.03^a^	14.48±1.14^a^
	Good (3)	93	8.98±0.99^a^	9.78±0.68^b^	0.22±0.03^a^	18.99±1.35^b^
	Fat (4-5)	27	8.74±1.71^a^	6.91±0.86^a^	0.18±0.05^a^	15.85±2.05^ab^
	P-value					0.038
Pregnancy status	Pregnant	105	9.23±0.79^a^	7.55±0.64^a^	0.29±0.03^a^	17.00±1.26^a^
	Non pregnant	96	7.63±0.79^a^	8.69±0.59^a^	0.31±0.03^a^	16.52±1.11^a^
	P-value					0.781
Pregnancy length	1^st^ trimester	53	8.20±1.34^a^	9.60±0.93^a^	0.27±0.05^a^	18.08±1.82^a^
	2^nd^ trimester	32	7.48±1.25^a^	8.50±0.88^a^	0.17±0.05^b^	16.12±1.69^a^
	3^td^ trimester	20	6.37±0.94^a^	6.60±1.15^a^	0.12±0.05^c^	13.05±1.74^a^
	P-value					0.431

^a,b,c^ In each column different letters (a, b) indicated significant difference between group (p<0.05). N=number.

**Table 3b:** Effects of non-ovarian factors on oocytes number and grade.

Factors		N	Average number of oocytes/ovary	Oocyte grades				Selected oocytes for IVEP. I and II (%)
				I	II	III	IV	
Breed	White Fulani	58	11.39±1.03^a^	3.63±0.28^a^	2.65±0.28^a^	2.42±0.58^a^	2.74±0.42^a^	6.27±0.60^a^ (55.12)
	Bokolo	20	11.07±1.93^a^	2.95±0.45^a^	3.27±0.63^a^	2.27±0.47^a^	2.42±0.69^a^	6.22±1.02^a^ (56.21)
	Red Fulani	31	10.79±1.82^a^	3.75±0.65^a^	2.55±0.40^a^	2.10±0.19^a^	2.21±0.53^a^	6.31±0.99^a^(58.41)
	Gudali	92	10.75±0.76^a^	3.53±0.26^a^	2.70±0.22^a^	2.23±0.15^a^	2.41±0.26^a^	6.23±0.45^a^ (58.00)
	P-value		0.809					1.000
Age (years)	3-5	55	12.23±0.95^a^	3.77±0.28^a^	2.94±0.27^a^	2.60±0.32^a^	2.91±0.37^a^	6.72±0.49^a^ (54.97)
	6-9	108	11.64±0.79^a^	3.79±0.28^a^	2.98±0.22^a^	2.35±0.19^a^	2.52±0.28^a^	6.77±0.46^a^(58.18)
	10-15	38	7.25±1.30^b^	2.46±0.49^b^	1.65±0.28^b^	1.41±0.26^b^	1.72±0.38^b^	4.12±0.75^b^(56.83)
	P-value		0.006					0.006
BCS	Thin (1-2)	81	9.59±0.82^a^	2.93±0.26^a^	2.44±0.25^a^	1.89±0.21^a^	2.31±0.34^a^	5.38±0.48^a^ (56.08)
	Good (3)	93	12.50±0.87^b^	4.34±0.31^b^	3.07±2.15^a^	2.49±0.23^a^	2.59±0.26^a^	7.42±0.49^b^ (59.36)
	Fat (4-5)	27	9.87±1.52^a^	2.55±0.41^a^	2.33±0.38^a^	2.41±0.43^a^	2.57±0.58^a^	4.89±0.75^a^ (49.54)
	P-value		0.034					0.03
Pregnancy status	Pregnant	105	12.05±0.85^a^	3.62±0.27^a^	2.98±0.24^a^	2.53±0.23^a^	2.90±0.32^a^	6.61±0.48 ^a^ (54.88)
	Non pregnant	96	9.99±0.75^b^	3.46±0.28^a^	2.48±0.19^a^	1.97±0.18^b^	2.08±0.24^a^	5.93±0.44 ^a^ (59.40)
	P-value		0.023					0.298
Pregnancy length	1^st^ trimester	53	11.33±1.27^a^	3.87±0.45^a^	2.82±0.33^a^	2.23±0.29^a^	2.41±0.39^a^	5.26±0.72^a^ (59.00)
	2^nd^ trimester	32	9.41±0.98^b^	3.47±0.42^a^	2.30±0.25^a^	1.92±0.29^a^	1.72±0.31^a^	5.76±0.59^a^(61.29)
	3^td^ trimester	20	7.37±1.26^b^	2.35±0.46^a^	1.85±0.33^a^	1.37±0.30^a^	1.80±0.50^a^	4.20±0.76^a^ (56.94)
	P-value		0.016					1.430

^a,b,c^ In each column different letters (a, b) indicated significant difference between group (p<0.05). N=number.

## Discussion

The mean age of the slaughtered cows at the NMSH was similar to that reported by Bah *et al*. (2010). However, the breed characteristics of the slaughtered cows in this study indicated that higher number of Mbororo breeds (White Fulani and Red Fulani) and Bokolo are being slaughtered at the NMSH than in the previous study. This is due to the fact that 50.24% of the slaughtered cows, mostly the Mbororo and Bokolo breeds came from the Mayo Rey Division, in the neighboring North Region. Zebu Gudali are more expensive than the Mbororo and this may explain the current demand.

The mean weight of Cameroonian zebu ovaries is comparable to that of Ankole zebu (4.6±2.3) in Uganda reported by Natumanya *et al*. (2008), but lighter than that of Friesian breed (10–19 g) and Swedish Red (10.2 g) obtained by Pierson and Ginther (1987a) and Rajakoski (1960), respectively. This difference may be due to the breed effect. In fact the mean live weight of slaughtered cows was lower compared to that of the Friesian (547-793 kg) (Laizeau, 2003). The disproportion between the right and left ovary was also reported by Rajakoski (1960) and Trigal *et al*. (2014). Indeed, studies of Pierson and Ginther (1987a) and Ginther *et al*. (2013) showed that ovulations are more frequent on the right ovary. This greater physiological activity on the right ovary would be responsible for the increase of its weight (Ginther *et al.*, 2013). The sizes of the ovaries in this study were lower than those of European breeds measuring 3 - 43 mm long, 19 - 23 mm wide and 13 - 19 mm thick. Previous studies have shown that the nutritional status, the breed, the presence of follicles and CL influence the size of the ovary and hence its weight (Pierson and Ginther, 1987b).

The positive association between the BCS and the thickness of the ovary could be explained by negative energy balance that impacts the general condition of the animals and therefore their ovaries (Diskin *et al.*, 2003).

The mean number of follicular population obtained is comparable to that reported by Carvalho *et al*. (2008) in Nellore zebu (16.7±1.6). Meanwhile the number of medium follicles is similar to that of Ankole zebu (Natumanya *et al.*, 2008) but is different from that of European breeds (Dominguez, 1995; Silva-Santos *et al.*, 2011). These variations are related to either breed or to the livestock’s environment.

The slicing technique enables the recovery of all ovarian oocytes present in all follicles regardless of their location in the ovarian cortex. Oocyte yield was comparable to that reported by Wang *et al*. (2007) in Holstein; however, it is higher than that reported by Armstrong (2001), Natumanya *et al*. (2008) and Abraham *et al*. (2012): 6.0±0.6; 4.05±0.77 and 3.33±1.03, respectively; and less than 22.3 and 66 oocytes reported by Carolan *et al*. (1992). These variations may be related to the breed. The oocyte quality index of this study being greater than one indicated that the overall quality of oocytes was average. However, oocytes with quality GI and GII were in the acceptable range of 30 to 60 oocytes for IVEP as suggested by Lucyna and Zdzisław (1984) and Natumanya *et al*. (2008).

Few studies have reported the effect of size and weight of ovaries on the ovarian follicular population, oocytes yield and quality in Sub-Sahelian zebu cattle (*Bos indicus*). The oocyte recovery rate increased significantly (P=0.000) with the weight and size of the ovary in accordance to findings in buffalo (Samad and Raza, 1999). Natumanya *et al*. (2008) also reported no influence of CL on oocyte recovery rate in Ankole cattle. Although, Ginther *et al*. (2013) reported a positive influence of CL on the number of ovarian follicles, they factored the presence of dominant follicles in their analysis which was not the case in this study. Greater follicular activity was reported on right ovaries carrying a dominant follicle and/or CL by Ginther *et al*. (2013).

BCS had a positive influence on follicular population in accordance with other findings (Dominguez, 1995; Alves *et al.*, 2014). In fact, cows with negative energy balance may have direct consequences on the hypothalamic-pituitary axis, by reducing the secretion of pituitary gonadotropins (FSH) responsible for follicular development (Diskin *et al.*, 2003). Basal folliculogenesis is essentially controlled by growth hormones such as Insulin Growth Factor-1 (IGF-1). During an energy deficit, there is deceleration of follicular growth due to a decrease in concentrations of IGF-1 (Lucy *et al.*, 1992). High plasma levels of IGF-1 resulting from improved nutrition, increases the sensitivity of granulosa cells to FSH stimulation (O’Callaghan and Boland, 1999). Ryan *et al*. (1994) also found a relationship between the blood concentration of IGF-1 and BCS: fat or thin animals presented low concentrations of IGF-1.

The effect of age on follicular population and ovary recovery rate as obtained in this study was also reported by Lucyna and Zdzisław (1984) and Fassi (2006). Armstrong (2001) showed that fertility as well as follicular population declines with age in all species. Contributing factors such as the deficiency of ovarian hormones, apoptosis of follicles and senility may be the main factors responsible for the depletion of ovarian reserve.

This study also demonstrated that the pregnancy status of cows in the first, second and third trimester of pregnancy did not affect the follicular population. Ginther *et al*. (1989) reported that there is a continuous emergence of waves of follicular growth every 8 to 10 days without dominance during pregnancy despite the continuous production of progesterone. But follicular size decreases with the age of the fetus (Dominguez, 1995).

Although the persistence of follicular waves were observed between the 4^th^ and 9^th^ month of pregnancy in our local zebu cows, no follicle with diameter greater than 6 mm was detected on the ovaries during the last three weeks of pregnancy (Ginther *et al.*, 1996). The presence of CL in pregnant cows has negative effect on the growth of follicles greater than 7 mm diameter after the 22^nd^ day of pregnancy (Pierson and Ginther, 1987b) due to the inhibition of the secretion of FSH by CL.

There was no influence of the local breeds on the quantity and quality of oocytes recovered. Similar findings were reported in Swedish Red and Swedish Holstein (Abraham *et al.*, 2012). Dominguez (1995) and Fassi (2006) noted that European breeds of cows gave more oocytes than local breeds. In concordance with our findings the quantity and quality of oocytes have been shown to decrease with age (Lucyna and Zdzisław, 1984; Natumanya *et al.*, 2008) and young cows remain the best choice to increase the production of good embryos. The effect of BCS and pregnancy on the quantity and quality of oocyte was also reported by Dominguez (1995) and Natumanya *et al*. (2008). Indeed, metabolic and hormonal changes not only affect follicular growth but also the growth of the oocytes (Monniaux *et al.*, 2009).

## Conclusion

This study indicated that the ovaries of local zebu breed raised in Cameroon have an average potential for IVEP. Future studies are required to perform IVM and IVF and must take into account certain factors to maximize success such as age, BCS, pregnancy status and ovary size.
